# Chemical Composition and Insulin-Resistance Activity of Arginine-Loaded American Cranberry (*Vaccinium macrocarpon* Aiton, Ericaceae) Leaf Extracts

**DOI:** 10.3390/pharmaceutics15112528

**Published:** 2023-10-25

**Authors:** Oleh Koshovyi, Inna Vlasova, Heleriin Laur, Ganna Kravchenko, Oksana Krasilnikova, Sebastian Granica, Jakub P. Piwowarski, Jyrki Heinämäki, Ain Raal

**Affiliations:** 1 Institute of Pharmacy, Faculty of Medicine, University of Tartu, Nooruse 1, 50411 Tartu, Estonia; oleh.koshovyi@gmail.com (O.K.); jyrki.heinamaki@ut.ee (J.H.); 2 Department of Pharmacognosy, National University of Pharmacy, 53 Pushkinska Str., 61002 Kharkiv, Ukraineannabk2014@gmail.com (G.K.);; 3 Microbiota Lab, Department of Pharmaceutical Biology, Faculty of Pharmacy, Medical University of Warsaw, Banacha 1, 02-097 Warsaw, Polandjakub.piwowarski@wum.edu.pl (J.P.P.)

**Keywords:** American cranberry, chemical composition, insulin resistance effect, L-arginine, leaf extract, *Vaccinium macrocarpon*, 3D printing

## Abstract

One of the key pathogenetic links in type 2 diabetes mellitus (T2DM) is the formation of insulin resistance (IR). Besides a wide selection of synthetic antidiabetic drugs, various plant-origin extracts are also available to support the treatment of T2DM. This study aimed to investigate and gain knowledge of the chemical composition and potential IR correction effect of American cranberry (*Vaccinium macrocarpon* Aiton) leaf extracts and formulate novel 3D-printed oral dosage forms for such extracts. The bioactivity and IR of L-arginine-loaded cranberry leaf extracts were studied in vivo in rats. The cranberry leaf extracts consisted of quinic, 3-caffeoylquinic (chlorogenic), *p*-coumaroylquinic acids, quercetin 3-*O*-galactoside, quercetin-3-*O*-glucoside, quercetin-3-xyloside, quercetin-3-*O*-arabino pyranoside, quercetin-3-*O*-arabinofuranoside, quercetin 3-*O*-rhamnoside, and quercetin-*O*-*p*-coumaroyl hexoside-2 identified by HPLC. In vivo studies with rats showed that the oral administration of the cranberry leaf extracts had a positive effect on insulin sensitivity coefficients under the insulin tolerance test and affected homeostasis model assessment IR levels and liver lipid content with experimental IR. A novel 3D-printed immediate-release dosage form was developed for the oral administration of cranberry leaf extracts using polyethylene oxide as a carrier gel in semi-solid extrusion 3D printing. In conclusion, American cranberry leaf extracts loaded with L-arginine could find uses in preventing health issues associated with IR.

## 1. Introduction

Diabetes (mainly types 1 and 2) is a global medical and social problem and is becoming a true challenge for health care. In developed countries, approximately 5–12% of the population suffers from diabetes, and it is predicted that the present value may increase up to 30–35% in the near future [1]. One of the key pathogenetic links of type 2 diabetes mellitus (T2DM) is the formation of insulin resistance (IR) syndrome, which triggers a cascade of disturbances in almost all metabolic links and leads to the formation of fatal complications that not only reduce the quality of a patient’s life but also shorten its average duration compared with patients without diabetes [1]. According to some modern ideas about the molecular mechanisms of IR pathogenesis, the condition is accompanied by the intensification of free radical oxidation. In addition, due to persistent hyperglycemia and a decrease in the inhibitory effect of insulin on lipolysis, deep disorders of lipid metabolism occur, resulting in the accumulation of lipids in the liver tissue and the development of atherogenic dyslipidemia [2,3].

Today, there is a wide selection of antidiabetic drugs, among which biguanide derivatives (such as metformin) are first-line drugs and sulfonylurea derivatives (e.g., glibenclamide) and some other synthetic antidiabetic drugs are classified as second-line therapies. In addition to synthetic antidiabetic drugs, some plant-origin medicines are quite widely used in T2DM drug therapy. For example, biguanide derivatives have several restrictions in their use: the adverse effect on the fetus, contraindications during pregnancy and lactation, and complications in the form of lactic acidosis. Sulfonylureas derivatives, in turn, possess the risk of changing the concentration of other drugs in the blood; therefore, they are used with caution in complex therapy [4].

In addition to synthetic antidiabetic drugs, some plant-origin medicines are quite widely used in T2DM drug therapy. For example, the tea mixture “Arfazetin” is an herbal drug product registered on the pharmaceutical market in Ukraine for T2DM drug therapy. The advantages of such natural medicines include a positive (high) safety profile and the possibility of using them as a part of complex therapy in combination with synthetic drugs. However, the well-known limitations of such tea mixtures are the lack of a convenient oral dosage form for consumption at home (for example, a decoction) and the lack of standardization of such tea mixtures. Therefore, it is advisable to develop new extracts and oral dosage forms through modern scientific achievements (including pharmaceutical 3D printing) to improve the use and efficacy of natural plant-origin medicines [5].

In the state-of-the-art literature, it has been proven that flavonoids prevent experimental hepatic steatosis, dyslipidemia, and IR, primarily through inhibition of hepatic fatty acid synthesis and enhancement of their oxidation [6]. Many flavonoids are also able to affect the levels of α-glycosidase, glucose cotransporter, and aldose reductase [7]. Lipid metabolism disorders are a key player in the pathogenesis of diet-induced IR and further T2DM [6,7].

American cranberry (*Vaccinium macrocarpon* Aiton) is especially rich in flavonoids (including anthocyanins), polyphenols, and other biologically active compounds, and consequently, it is associated with a number of beneficial health effects [8,9]. Previous studies have shown that the extracts obtained from the leaves of *Vaccinium* genus species are promising for formulating hypoglycemic and hypolipidemic medicines. More specifically, such extracts were obtained, e.g., from bilberry leaves [10,11], highbush blueberry leaves [12], and bearberry leaves [13,14]. Moreover, the extracts of American cranberry leaves were proven to have hepatoprotective effects [8]. Therefore, the extracts prepared from American cranberry leaves may also be promising agents for IR correction.

The aim of the present study was to investigate and gain knowledge of the chemical composition and hypoglycemic/hypolipidemic activity of novel extracts obtained from *V. macrocarpon* Aiton leaves, including L-arginine-loaded extracts. The pharmacological activity of the extracts was studied in vivo in rats. An aqueous gel formulation loaded with the cranberry leaf extract for semi-solid extrusion (SSE) 3D printing and for preparing novel 3D-printed oral dosage forms for such plant extracts has also been developed.

For the first time, it was proposed to use the leaves of American cranberries to create extracts with hypoglycemic and hypolipidemic activity. Beforehand, only fruits were commonly used in pharmaceutical and medicinal practices. Leaves are usually wasted while cultivating the plant. Also, the new American cranberry leaf extract modified with arginine was proposed for the first time. It is more effective than the native one. The composition of the promising aqueous polyethylene oxide (PEO) gel loaded with cranberry extracts was developed, which is suitable for SSE 3D printing of dietary supplements for supporting the treatment of metabolic disorders related to T2DM.

## 2. Materials and Methods

### 2.1. Chemicals and General Experiments

Deionized water was produced using a Millipore Simplicty UV station (Merck Millipore, Burlington, MA, USA). Acetonitrile, formic acid, and ethanol were purchased from VWR (Radnor, PA, USA). The following chemicals were used: chlorogenic acid, rutin, gallic acid (Carl Roth, Karlsruhe, Germany), L-arginine, Tween-80, and aluminum chloride (Sigma-Aldrich, Sant Louis, MI, USA), eumulgin SMO 20 (polyethylene glycol 40–hydrogenated castor oil, Polysorbate 80) (LOT S721580003, Cognis, France), quercetin (Borschagovsky CPP, Kyiv, Ukraine), and fructose (LLC “Ukrhimsyre”, Kharkiv, Ukraine). Blood glucose, high-density lipoprotein cholesterol (Ch-HDL), and low-density lipoprotein cholesterol (Ch-LDL) (Felitis-Diagnostics, Ukraine) Insulin (DRG, Germany) and triacylglycerols (TAG, Lachema, Czech Republic) were determined in blood serum using the standard sets of reagents. Also, metformin (Teva Pharmaceutical Industries Ltd., Israel) has been used. The chemical standards used for HPLC analysis were previously isolated and identified in the Department of Pharmacognosy and Molecular Basis of Phytotherapy, Medical University of Warsaw, Poland. The software of an SSE 3D printer is Repetrel, Rev3.083_K, Hyrel 3D, USA.

### 2.2. Plant Material

*V. macrocarpon* Aiton leaves were harvested in August 2020 in Kyiv (Pereyaslav suburbs 50.10314334026342, 31.46151900698126). The identity of the plant was established by Professor Tetiana Gontova, D.Sc. [15]. Voucher specimens were deposited in the Department of Pharmacognosy (National University of Pharmacy, Kharkiv, Ukraine, No. 592–594). The raw material was dried at room temperature in a well-ventilated area for ten days and stored in paper bags [16]. *V. macrocarpon* leaves correspond to the established parameters of standardization [17].

### 2.3. Preparation of Extracts

For preparing L-arginine-loaded American cranberry leaf extract (PE + Arg), 250 g of dried *V. macrocarpon* leaves [17] ground to a particle size of 1–2 mm were placed in an extractor and macerated with 1.25 L of ethanol:water mixture (1:1, *v*/*v*) overnight at room temperature. The extraction was repeated once with new portions of the solvent (0.75 L). The resulting extracts were combined, settled for 24 h, and filtered through a folding filter. The first part of a liquid extract (500 mL) was evaporated using a rotary vacuum evaporator to form a dry extract (PE). The yield of the dry extract was 24.2%.

L-Arginine (8.34 g) was added to the second part of a liquid extract (500 mL) in a three-fold equimolar amount to the total phenolic compounds in terms of gallic acid. The resulting solution was kept for one day at room temperature and evaporated using a rotary vacuum evaporator to a dry extract (PE + Arg). The yield of the dry extract was 38.4%.

### 2.4. HPLC-DAD-MS Analysis of Extracts

The HPLC-DAD-MS analysis of PE and PE + Arg extracts was performed using Ultimate 3000 RS system (Dionex, Sunnyvale, CA, USA) coupled with an ion-trap mass spectrometer Amazon SL (Bruker Daltonik, Bremen, Germany). The separation was carried out with a Kinetex XB-C_18_ column (150 mm × 2.1 mm × 1.7 μm, Torrance, CA, USA). The column was eluted with 0.1% formic acid in deionized water (A) and 0.1% formic acid in acetonitrile (B). The gradient program was used as follows: 0 min—1%B, 60 min—26%B. The flow rate was 0.3 mL/min, and the column temperature was kept at 25 °C. The eluate was introduced directly to the ESI source of the mass spectrometer. The ESI source parameters were nebulizer pressure 40 psi; dry gas flow 9 L/min; dry temperature 135 °C; and capillary voltage 4.5 kV. The compounds were analyzed in the negative and positive ion modes. The MS/MS mode was active, and the most abundant ion in the recorded spectrum was subjected to fragmentation. The signals obtained in the MS/MS spectrum were used for further fragmentation whenever possible with Smart Frag mode. The UV-Vis spectra of detected compounds were monitored from 200 to 450 nm by using a DAD device [12,18].

### 2.5. Assay of Main Phytochemicals

Spectrophotometry was used for the quantitative determination of hydroxycinnamic acid derivatives, flavonoids, amino acids, and total phenolic compounds in the extracts in terms of a dry residue. The optical density of solutions was measured with a Specol 1500 spectrophotometer (Thermo Fisher Scientific, Basel, Switzerland). The content of hydroxycinnamic acids was determined in terms of chlorogenic acid directly at 327 nm and at 525 nm after reaction with hydrochloric acid, sodium nitrite, and sodium molybdate [12,19]. The total flavonoid content was determined in terms of rutin (at a wavelength of 417 nm) and hyperoside (at a wavelength of 425 nm) after the formation of the complex with aluminum chloride [20,21]. The content of total phenolic compounds was obtained in terms of gallic acid directly at 270 nm and after the reaction with Folin & Ciocalteu′s phenol reagent (λ = 765 nm) [22]. The content of amino acids was analyzed after reaction with ninhydrin solution in terms of leucine (λ = 573 nm). For statistical validity, the experiments were performed five times [23,24].

### 2.6. The Pharmacological Activity of Extracts

For in vivo studies, 3-month-old male outbreed white rats, which were standardized by body weight of 190 ± 10 g, were used. The rats were kept in the Vivarium of the Educational and Scientific Institute of Applied Pharmacy, National University of Pharmacy (NUPh), Kharkiv, Ukraine. The rats of all groups were fed with standard chow, and intact control animals had free access to water. At the same time, the rats were watered with a 20% fructose solution ad libitum for 5 weeks in order to enrich the diet with fructose (high-fructose diet, HFD) and model IR [11,12]. As a primary positive control drug, metformin was used, which is considered the drug of choice for T2DM treatment, and it is also the main representative of a biguanide group with a well-studied mechanism of hypoglycemic action. As the second reference medication, a plant officinal tea mixture called "Arphazetin,” officially registered as a medicinal product in Ukraine, was used. The rats were randomly divided into the following experimental groups (n = 6): group 1 (IC)—intact animals without any treatment; group 2 (IR)—animals with experimental IR; group 3 (IR + PE)—animals with experimental IR that were intragastrically administered with PE in dose 200 mg/kg bw begining from the 5th week of experiment during 2 weeks; group 4 (IR + PE + Arg)—animals that were intragastrically administered with PE + Arg according to the same scheme (group 3) in dose 200 mg/kg bw; group 5 (IR + Arg)—animals with experimental IR that were intragastrically administered with L-arginine (Sigma-Aldrich, USA) according to the same scheme (group 3) in dose 100 mg/kg bw; group 6 (IR + Arph)—animals with experimental IR that were intragastrically administered with tea infusion “Arphazetin” (PJC “Liktravy”, Zhytomyr, Ukraine) according to the same scheme (group 3) in dose 18 mL/kg bw; group 7 (IR + Met)—animals with experimental IR that were intragastrically administered with metformin according to the same scheme (group 3) in dose 100 mg/kg bw.

The animals from all groups had their body weight recorded each week of the experiment. The IR development and treatment were monitored by conducting the oral glucose tolerance test (OGTT), the insulin tolerance test (ITT), and recalculating the HOMA-IR index. OGTT was performed at the end of the 7th week of the experiment after 12 h of fasting [25]. Blood samples were taken by incision of the gums in rats [26], and blood glucose concentrations were determined with the help of a “One Touch Select” glucometer (LifeScan, Malvern, PA, USA). Blood glucose concentration was determined at “time 0” and, subsequently, a glucose solution in dose of 3 g/kg bw (LLC “Istok-Plus”, Zaporizhzhia, Ukraine) was administered to the rats intragastrically. Blood samples were collected at regular intervals of 30, 60, 90, and 120 min. For determining OGTT, the area under the curve (AUC) was calculated using a trapezoidal method [Glucose area under the curve during oral glucose tolerance test] as an index of glucose intolerance [27].

The ITT was carried out 48 h after the OGTT, and the results were presented in the form of an insulin sensitivity coefficient. This coefficient shows the percentage of reduction in blood glucose every 30 min up to 120 min after the intraperitoneal injection of exogenous insulin (“Novo Nordisk”, Bagsværd, Denmark) in dose 1 U/kg bw relative to basal glycemia (after overnight fasting). At the end of the 7th week of the experiment and after overnight fasting, the rats were sacrificed by decapitation under ketamine anesthesia (“Biovet Pulawy”, Pulawy, Poland). Blood samples were collected to obtain blood serum. Livers were removed, perfused with an ice-cold 0.9% sodium chloride solution, and 10% liver homogenates (10 mM Tris-HCl buffer 7.4) were prepared.

Fasting blood glucose (FBG) and immunoreactive insulin (IRI) concentrations were determined using commercially available kits (LLC “Felicit Diagnostics” in Ukraine and “DRG” in Germany, respectively). Then, a homeostasis model assessment (HOMA-IR) index was calculated using a special web-based calculator at the Oxford website (https://www.dtu.ox.ac.uk/homacalculator/ (accessed on 12 October 2023) HOMA-IR = (I0 × G0)/22.5, where G—FBG in mmol/L). To evaluate a lipid metabolism state in blood serum, the content of triacylglycerols (TG), total cholesterol (Ch), cholesterol-HDL (Ch-HDL), and cholesterol-LDL (Ch-LDL) was determined using commercially available kits (LLC “Felicit Diagnostics”, Dnipro, Ukraine). In liver homogenates, the content of TG, diacylglycerols (DG), total phospholipids (TPhL), Ch, and free fatty acids was determined. Lipids were extracted according to the method described by Folch et al. [28]. The chloroform phase was collected and dried under nitrogen gas at 37 °C. The lipids were redissolved in chloroform/methanol (1:2, *v*/*v*) and applied to thin-layer chromatography (TLC) plates. For TLC, hexane/diethyl ether/acetic acid (80:20:2, *v*/*v*) was used as a solvent system. The appropriate standards were applied to each TLC plate for quantification. The gel spots containing lipids were scraped, and the contents of lipids in chromatographic fractions were determined by the method of Marsh and Weinstein [29]. The content of protein in the samples was determined according to Lowry’s Miller modification [30].

All animal tests were performed according to the Protocol of Amendment to the European Convention for the Protection of Vertebrate Animals used for Experimental and other Scientific Purposes (Strasbourg, 1986, as amended, 1998), the Law of Ukraine “On protection from cruelty to animals” (dated 15 December 2009, No. 1759-VI), and the European Union Directives 2010/10/63 EU about animal experiments. The protocol for the animal studies was subjected to the Ethics Committee for Animal Experiments of the NUPh (Protocol #3 from 10 September 2020; Approval #3/10092020).

### 2.7. Preparation of Gels Loaded with Cranberry Extracts for 3D Printing

The aqueous gels of PEO (MW approx. 900,000, Sigma-Aldrich, USA) at concentrations of 12% and 15% were used as a formulation platform for the SSE 3D printing of PE and PE + Arg. For preparing such gels, PEO (1.2 g, 1.5 g) was dissolved in distilled water (10 mL) approximately for at least 13–15 h at ambient room temperature to form a viscous gel [31,32]. Eumulgin SMO 20 (polyethylene glycol 40–hydrogenated castor oil, Polysorbate 80) was used to enhance the release of cranberry extracts from the 3D-printed preparations [32,33]. The cranberry extracts (1.0 g) and eumulgin as a surface-active agent (1.5 g) were added to 12% and 15% PEO gels. The viscosity of gels was determined with a Physica MCR 101 rheometer (Anton Paar, Austria) using a cone-plate geometry. The measurements were carried out at room temperature (21–25 °C). The viscosity measurements were performed by using a rotational shear test at the different shear rates.

### 2.8. Three-Dimensional Printing of Cranberry Extracts

The PEO gels loaded with cranberry extracts were directly printed using a bench-top SSE 3D printing system (System 30 M, Hyrel 3D, Norcross, GA, USA). The printing head consists of a steel syringe with a plunger connected to a stepper motor (the stepper motor moves the plunger up or down and pushes the content in the syringe out). A blunt needle (Gauge, 21G) connected to a syringe serves as a printing nozzle. The printing head (a syringe with a nozzle) was not heated. During SSE 3D printing, a printing head moved at a set speed on the X-Y axis (=printing speed) and extruded printing material at a specified speed through a nozzle system (=extrusion speed) onto a thermostated printing plate. The printing plate temperature was set at 30 °C. Following every printed layer, a printing plate was lowered by a predefined distance (layer height), thus allowing a printing head to create another layer of material on top of a printed object. The software of an SSE 3D printer controls the temperature of the printing head and plate, the moving speed of the printing head, the gel extrusion rate, and other settings. The printing head speed used was 0.5 mm/s. A total of 8 layers were printed for the model lattices and 5 layers for the round-shaped disc preparations.

For verifying 3D printing quality, a model 4 × 4 grid lattice was designed with Autodesk 3ds Max Design 2017 software (Autodesk Inc., San Francisco, CA, USA). The dimensions for a square-shaped 3D lattice were 30 × 30 × 0.5 mm. The evaluation of 3D printability was based on the printed lattice weight and area measurements. The theoretical surface area of a square-shaped 3D lattice (324 mm^2^) was compared with the corresponding areas of experimental 3D-printed lattices [31,33]. A round-shaped disc preparation (20 mm in diameter) was designed using FreeCAD software (vers. 0.19/release date 2021) [34].

The 3D-printed PEO lattices and round-shaped disc preparations were weighed with an analytical scale (Scaltec SBC 33, Scaltec, Germany) and subsequently photographed. The photographs were analyzed with ImageJ (National Institute of Health, Bethesda, MD, USA) image analysis software (version 1.51k). With the 3D-printed lattices, the experimental value obtained for a surface area was compared with the corresponding theoretical value of a designed lattice.

### 2.9. Statistical Analysis

The statistical properties of random variables (with an n-dimensional normal distribution) are given by their correlation matrices, which can be calculated from the original matrices. A statistical assessment of the data was performed using MS Excel (Microsoft Excel 2016, version 16.0, Microsoft Corporation, Redmond, DC, USA). P values less than 0.05 were considered statistically significant [35].

## 3. Results

### 3.1. Phytochemical Analyses of Cranberry Leaf Extracts

**Figure 1:**
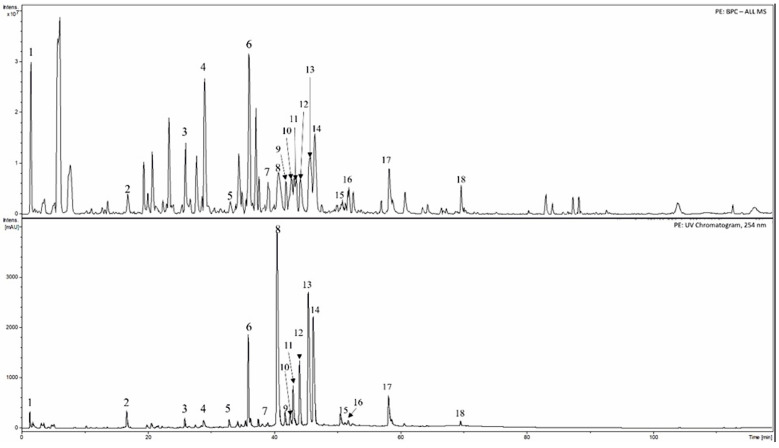
Typical HPLC-DAD-MS chromatograms of cranberry leaf extract: 1. quinic acid; 2. 3-*O*-caffeoylquinic acid (chlorogenic acid); 3. unknow compound; 4. (+)-catechin; 5. proanthocyanidin A type; 6. unknow compound; 7. unknow compound; 8. quercetin 3-*O*-galactoside; 9. quercetin-3-*O*-glucoside; 10. procyanidin dimer A2 type; 11. quercetin-3-*O*-xyloside; 12. quercetin-3-*O*-arabino pyranoside; 13. quercetin-3-*O*-arabino furanoside; 14. quercetin 3-*O*-rhamnoside; 15. quercetin-*O*-*p*-coumaroyl hexoside-1; 16. kaempferol 3-*O*-rhamnoside; 17. quercetin-*O*-*p*-coumaroyl hexoside-2; 18. quercetin derivative.

**Figure 2:**
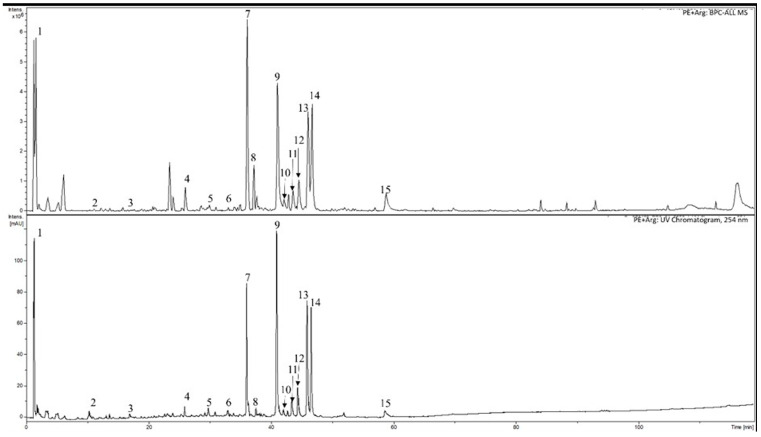
Typical HPLC-DAD-MS chromatogram of the cranberry leaf extract preparation with arginine: 1. quinic acid; 2. unknown compound; 3. 3-caffeoylquinic acid (chlorogenic acid); 4. unknown compound; 5. *p*-coumaroylquinic acid; 6. unknow compound; 7. unknow compound; 8. unknow compound; 9. quercetin 3-*O*-galactoside; 10. quercetin-3-*O*-glucoside; 11. quercetin-3-xyloside; 12. quercetin-3-*O*-arabino pyranoside; 13. quercetin-3-*O*-arabino furanoside; 14. quercetin 3-*O*-rhamnoside; 15. quercetin-*O*-*p*-coumaroyl hexoside-2.

The extracts of cranberry leaves (PE and PE + Arg) are dry powders with a light brown color and a weak, specific smell. The HPLC-DAD-MS was used to identify some main phenolic substances in the cranberry extracts (Table 1, Figure 1 and Figure 2). The results of spectrophotometric analysis are shown in the Table 2.

### 3.2. The Pharmacological Activity of Extracts

**Figure 3:**
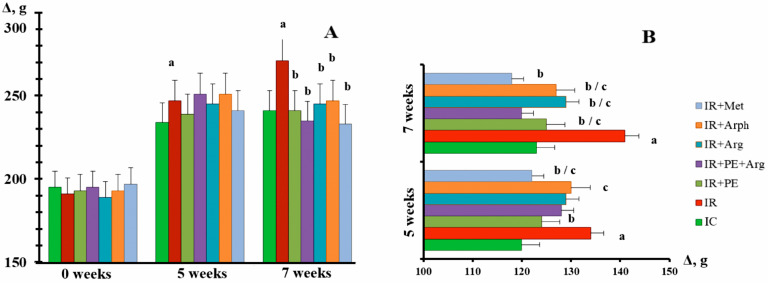
Mean body weight of the rats in the experimental groups at the 5th week from the zero point and after 2 weeks of treatment. (**A**)—absolute weight (g); (**B**)—body weight growth (%). a—indicates a significant difference relative to the IC group (*p* ≤ 0.05). b—indicates a significant difference relative to the IR group (*p* ≤ 0.05). c—indicates a significant difference relative to the IR + PE + Arg group (*p* ≤ 0.05).

The rats in all groups, which were selected for HFD-stimulated IR development, had a similar initial body weight, and at the end of the 5th week of HFD before the treatment, they showed weight gain, which was significantly higher as compared with the IC group (Figure 3A). Accordingly, body weight growth (as a percentage of body weight) was significantly higher with the rats in the IR group as compared with the rats in the IC group. Further in the experiment, IR group rats had a significantly greater increase in mean body weight from week 5 to week 7 (Figure 3A). At the same time, the rats that were fed with HFD and given the medicines showed a tendency towards a decrease in body weight.

**Figure 4:**
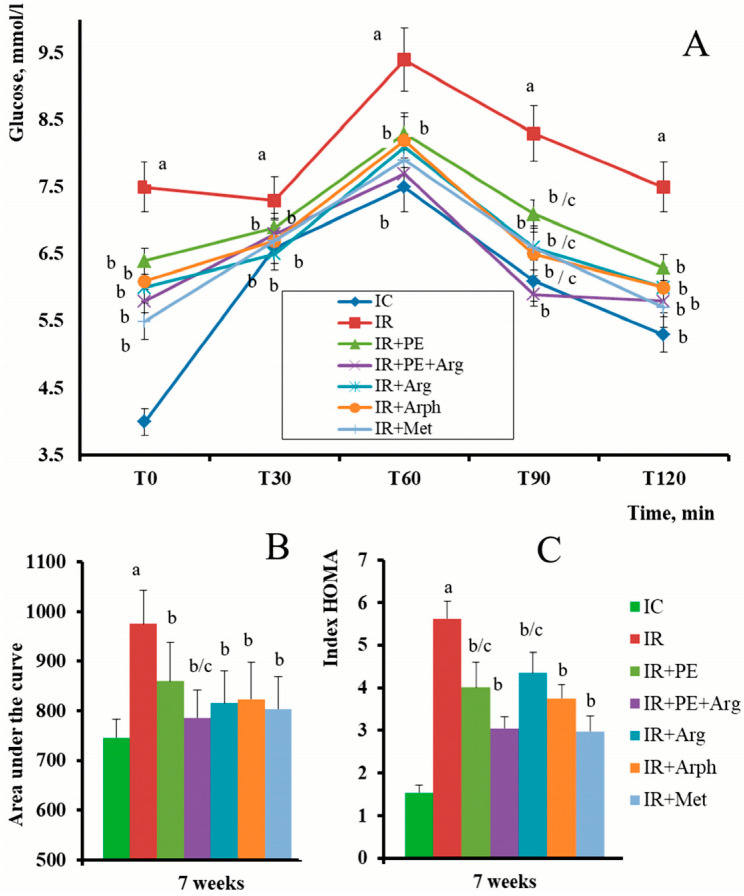
Hypoglycemic effect in the experimental groups of rats at the 7th week from the zero point and after 2 weeks of treatment. (**A**)—OGTT; (**B**)—AUC data was calculated for OGTT (mmol/Lx120 min; (**C**)—HOMA-IR. a—indicates a significant difference relative to the IC group (*p* ≤ 0.05). b—indicates a significant difference relative to the IR group (*p* ≤ 0.05). c—indicates a significant difference relative to the IR + PE + Arg group (*p* ≤ 0.05).

The OGTT conducted after 2 weeks of the administration of antidiabetic drugs demonstrated the positive dynamics in IR correction. FBG in the IR group was significantly higher compared with the IC group, which remained practically at the same level from the beginning of the experiment. The administration of PE + Arg and metformin reduced basal glycemia (compared with the IR group) by 22.6% and 26.7%, respectively (Figure 4A). However, the main aim of this test was to evaluate the tolerance to glucose in dynamics. A significant difference in glucose tolerance between the rats in an IR group compared with the rats in an IC group has been found (Figure 4A). After a “glucose load”, glycemia in the rats of an IR group increased by 18.6% compared with the rats of an IC group, and this increase was found even after 120 min (Figure 4A). The administration of PE + Arg, PE, and positive control drugs clearly prevented the increase in glycemia in the rats. For example, at the time point of 90 min, the administration of PE + Arg decreased glycemia by 28.9%, “Arphazetin” by 21.6%, and metformin by 20.5% compared with the IC group. The present results showed that the treatment of rats with PE + Arg exceeded the OGT effect of “Arphazetin”, and showed an even comparable OGT effect to the administration of metformin in the OGTT. This was also demonstrated by the AUC calculation based on the results of the OGTT (Figure 4B). Accordingly, the AUC value for the rats in the PE + Arg group was 786 mmol/L × min, which was 19.4% less than the AUC value obtained with the rats in the IR group and even 4.8% less than the AUC for the rats in the IR + Arph group.

As shown in Figure 4C, the HOMA-IR index for the rats fed with HFD (IR group) was significantly higher compared with the corresponding index for healthy animals (IC group). It is evident that the administration of PE and PE + Arg to rats partially restores the insulin concentration and, consequently, the value of HOMA-IR to control levels, which is comparable with reference medications (Figure 4C).

**Figure 5:**
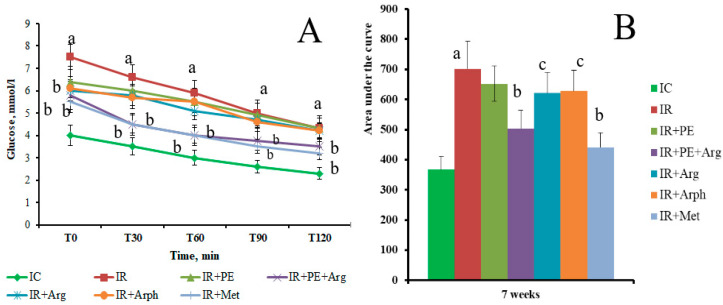
The insulin sensitivity of rats in the experimental groups at the 7th week from a zero point and after 2 weeks of treatment. (**A**)—ITT; (**B**)—AUC values calculated for ITT (mmol/Lx120 min). a—indicates a significant difference relative to the IC group (*p* ≤ 0.05). b—indicates a significant difference relative to the IR group (*p* ≤ 0.05). c—indicates a significant difference relative to the IR + PE + Arg group (*p* ≤ 0.05).

The sensitivity of peripheral tissues to insulin action was studied with the model of ITT. As shown in Figure 5, the ITT results indicate the development of IR in the rats of an IR group compared with the rats of an IC group. With the rats administered with IR + PE + Arg, the reduction of glucose level during the ITT was higher (*p* < 0.05) compared with the rats in the IR group. However, the difference in the reduction of glucose level was not statistically significant between the rats administered with IR + PE + Arg and the rats in the IC group. Interestingly, there was no statistically significant difference in glucose levels between the rats in the IR + Met group (Figure 5B). As seen in Figure 5, a significant decrease in insulin sensitivity was found in the rats of the IR group when conducting ITT. In the IR group, the glucose concentration decreased by only 19.7%, which is 27.4% less than in the IC group after 30 min from a zero point. As for reference medications, the insulin sensitivity coefficient increased and reached 31.9 and 41.2%, respectively. In support of this view, insulin sensitivity coefficients decreased under the ITT test, and the HOMA-IR levels were found to be higher in the rats of an IR group as compared with the rats of an IC group. PE + Arg supplementation partially restored the insulin sensitivity coefficient and HOMA-IR to control levels.

Table 3 summarizes the TG, total Ch (TCh), and Ch-LDL levels in the blood serum of rats determined at the end of the 7th week of the study. The results show that the present blood serum indices of lipid metabolism in the rats of an IR group increased by 38.2% (TG), 54.1% (TCh), and 50.1% (Ch-LDL) as compared with the levels in the rats of an IC group. After administering PE + Arg for 2 weeks under HFD, the concentrations of the first two indices were only 8.2% (TG) and 10.1% (TCh) higher than the levels of the corresponding indices in healthy rats. As shown in Table 3, the use of metformin from the 5th week of the experiment resulted in an increase of 11.4% of TG and a decrease of 2.3% of TCh as compared with the rats in an IC group. The administration of PE and PE + Arg significantly increased the TG and TCh levels in the rats. After seven weeks of HFD, a statistically significant increase in blood serum content of TG, TCh, and Ch-LDL in the rats was observed, while the level of Ch-HDL decreased, which reflects significant metabolic changes in the liver.

Feeding the rats with a diet enriched with fructose is accompanied by significant changes in the lipid spectrum of liver tissue. The results shown in Table 3 suggest that a significant increase in DG, TG, Ch, and FFA content accompanied by a decrease in PL content is evident in the liver of the rats in the IR group. The treatment of the rats with PE + Arg protected against an increase in TAG and DAG content and significantly increased the PL level in the rats. Thus, a significant decrease in the PL content by 1.39 times was observed, while the general content of lipids increased due to the growing content of the TG, FFA, and Ch by 1.19, 1.54, and 3.34 times, respectively.

The administration of PE and PE + Arg had a positive effect on the liver lipid content in the rats with an experimental IR. Thus, a significant increase in the PL content was observed in the rats, and the administration of PE + Arg was found to normalize the PL content. In addition, the administration of PE + Arg and PE reduced the content of TG, DG, and FFA, and with the rats in the IR + PE + Arg group, the content of TG decreased close to the level found with the rats in an IC group. As seen in Table 3, the FFA content in the rats of an IR + PE + Arg group was also significantly lower compared with that observed in the rats of an IR group. It is worth mentioning that the positive control medications given to the rats showed the expected effect on liver lipid metabolism, thus the harmful IR impact is decreasing.

### 3.3. Formulation of the Gels and 3D-Printed Dosage Forms of Cranberry Extracts

The aqueous PEO gels loaded with cranberry leaf extracts (1.0 g of the extract in 10 g of the gel) were brown viscous masses with a characteristic smell. The average viscosity of the 12% and 15% PEO gels loaded with cranberry extracts (10%) was 257033 cP and 344800, respectively. The viscosity of the gels was determined at a speed of 0.01 RPM and a shear rate of 0.020 1/s.

**Figure 6:**
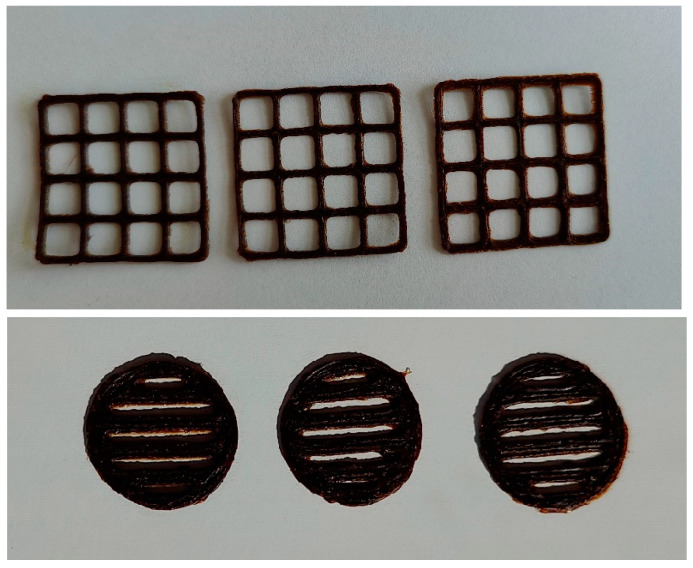
Photographs of the semisolid extrusion (SSE) 3D-printed lattices and round-shape discs of aqueous 12% PEO gel with a cranberry extract.

Based on the results of viscosity measurements and preliminary SSE 3D printing tests, the printing head speed of choice for the present aqueous PEO-PE gels was found to be 0.5 mm/s. The operating parameters for the SSE 3D printing of aqueous PEO gels were recently investigated and optimized by Viidik et al. [31]. Those results are relied on in the development of SSE 3D printing for the gels loaded with cranberry extract. Since the 3D printing properties of 15% aqueous PEO gels with PE were not satisfactory, the 12% PEO-PE gel for the subsequent SSE 3D printing experiments were tested. Figure 6 shows the experimental SSE 3D-printed lattices and round-shaped discs loaded with the present cranberry extract.

The feasibility of the aqueous PEO-PE gels for SSE 3D printing was verified by printing standard-size square-shaped 3D lattices with dimensions of 30 × 30 × 0.5 mm. The average weight of the 3D lattices and round-shape discs was 213.3 ± 22.9 mg and 174.8 ± 10.3 mg, respectively. The surface area of the 3D lattices ranged from 342 to 390 mm^2^, with an average area of 363.7 ± 39.6 mm^2^. The average S _practical_/S _theoretical_ ratio for the 3D lattices was 1.12.

## 4. Discussion

A total of 16 phenolic substances were identified in the cranberry leaf extracts. The substances are presented with 1 catechin, 2 hydrocinnamic acids, 2 procyanidins, 8 quercetin glycosides, and 1 kaempferol glycoside. It is worth mentioning, however, that one of the quercetin derivatives was not able to be fully identified. The present results are in accordance with our previous findings [8], showing that quercetin glycosides are the predominant flavonoids in the cranberry leaf extracts.

In the state-of-the-art literature, the great majority of publications have used and reported American cranberry fruits and their products, and only a few studies have reported the composition, properties, and medicinal use of leaves. The major active substances found in cranberry fruits are tannins, flavonoids, pectins, organic acids (ursolic, chinic, citric, benzoic, and others), ascorbic acid, sugars (glucose and fructose), and micro- and macroelements [9,33,36,37,38]. In general, *Vaccinium* genus fruits contain three classes of flavonoids, namely flavonols, anthocyanins, and proanthocyanidins [38]. The results showed that *V. macrocarpon* Aiton leaves and its extracts also present flavonols, mainly quercetin derivatives, and proanthocyanidins. Like fruits, the present leaves also contain hydrocinnamic acids and chlorogenic acid as predominant substances [36]. In our present study, the qualitative composition of the leaf extracts did not differ significantly from the composition reported in the previous studies [8,17]. Based on the different quantitative content of biologically active substances in cranberry fruits and leaves, especially flavonoids, a leaf advantage is expected.

The amounts of flavonoids and hydroxycinnamic acids in the extracts decreased by 1.6 and 1.5 times, respectively. This is obviously due to the addition of L-arginine, which leads to a conjugation with these substances. In our previous study, it was proven that in the corresponding conditions, hydroxycinnamic acids conjugate with amino acids [12]. The total phenolic compound content in the modified extract was 2.5 times less compared with the content of such compounds in the primary extract. Therefore, it is evident that the inclusion of L-arginine leads to physical and chemical changes in the modified leaf extract since the color of the extract became darker and the solubility in water improved. The changes in solubility may have an influence on the oral bioavailability of the extract.

Today, IR is one of the most common metabolic disorders that can gradually lead to a series of diseases, such as T2DM, non-alcoholic fatty liver disease, and cardiovascular diseases. Metabolic disorders that are accompanied by IR include hyperglycemia, glucose tolerance in peripheral tissues, oxidative stress development, dyslipidemic disorders, and proatherogenic state development [39,40]. The causes of IR are multi-factorial and not entirely understood.

According to the literature, diets enriched with fructose tend to cause the development of IR in rats, and such conditions are typically accompanied by body weight gain [40,41,42]. Such body weight gain in the rats of an IR group has also been observed (Figure 3). The development of IR in rats was accompanied by elevated FBG and impaired cell sensitivity to insulin action. This was indicated by the increase in the HOMA-IR index and the results of OGGT and ITT (Figure 4 and Figure 5). Interestingly, the administration of PE + Arg significantly reduced (hindered) the weight increase in the rats compared with the weight trend of the rats in an IR group. It is evident that this effect is mediated by diminished IR development.

The content of phenolic compounds, such as derivatives of hydroxycinnamic acids, flavonoids, and quercetin glycosides, most likely explains the hypoglycemic effect of PE and PE + Arg, and this is obviously due to their capacity to improve the sensitivity of cells to insulin [43]. Numerous in vitro, in vivo, and clinical studies have reported that plant-origin compounds (alone or in combination) can act as prospective therapeutic agents for the treatment of metabolic diseases accompanied by IR. Moreover, such plant-origin compounds reveal good results by minimizing complications [44,45]. The effects of plant-origin compounds are mediated by the regulation of enzyme activity and, in turn, by the regulation of signal transduction. For example, it has been shown that quercetin activates adenosine monophosphate kinase (AMPK) in skeletal muscles, which stimulates membrane binding of Akt (serine/threonine protein kinase B) and glucose transporter (GLUT4) receptors [46]. Moreover, quercetin stimulates an insulin-dependent AMPK pathway in other tissues, which is analogous to metformin activity [47]. In addition, flavonoids are potent antioxidants and capable of protecting cells from oxidative stress, including pancreatic cells [48].

The liver is the organ that plays a leading role in regulating the homeostasis of glucose and lipid metabolism. It has been demonstrated that liver ectopic lipids are associated with hepatic IR and trigger IR development in different organs and tissues, which cause metabolic diseases accompanied by a fatty liver, such as T2DM [6]. Hepatic IR is caused by DG-mediated activation of protein kinase C epsilon (PKCε), which is the predominant PKC isoform activated in the liver and has a high affinity for DG [49]. Hepatic DG content might be the best predictor of hepatic IR. Reducing hepatic lipid accumulation could be an effective way to improve hepatic IR. It has been shown that PKCε activates insulin receptor tyrosine kinase activity by inhibiting phosphorylation [6].

It is well known that excess fructose flow into the liver leads to a significantly enhanced rate of de novo lipogenesis and triglyceride synthesis, driven by the high flux of glycerol and acyl portions of TG molecules from fructose catabolism. This appears to be mediated by reduced insulin receptor and insulin receptor substrate 2 (IRS2) expression and increased protein-tyrosine phosphatase 1B (PTP1b) activity. On the other hand, the knockdown of ketohexokinase (KHK), the rate-limiting enzyme of fructose metabolism, is shown to increase insulin sensitivity [50].

Apparently, a decrease in the FFA and TG contents is mediated by suppression of the fatty acid synthase activity under plant polyphenol impact [51]. In addition, polyphenols could prevent the accumulation of FFA and TG in liver cells by enhancing fatty acid β-oxidation [52]. The administration of PE and PE + Arg had a positive effect on the lipid content in the liver of rats with IR (Table 3). Our results suggest that HFD lasting for seven weeks not only tends to induce hyperinsulinemia under IR but is also associated with increased hepatic lipogenesis, which may explain the dyslipidemia. Recently, Zhang et al. [53] reported that flavonoids may inhibit the expression of fatty acid synthase (FAS) in the liver by stimulating AMPK activity in hepatocyte cells, thus reducing fatty acid synthesis in the liver and fat accumulation. In addition, the activity of acetyl-CoA carboxylase and FAS may be inhibited [54].

According to the literature, the administration of L-arginine as an individual supplement or in combined therapy stimulates insulin sensitivity (considering that NO production is strictly associated with insulin resistance) and affects glucose and insulin homeostasis [55,56]. L-Arginine plays a crucial role in the pathogenesis of diabetes due to its activity against dysfunction of the vascular endothelium. The key factor regulating the tone of the vascular endothelium is the most important physiological vasodilator–nitrogen monoxide. This mediator is formed from arginine under the action of the Ca^2+^-dependent enzyme endothelial NO-synthase (eNOS) [12]. Previous research shows that using arginine in extracts’ modification increases the hypoglycemic and hypolipidemic activity of *Vaccinium corymbosum* L. [12], *Vaccinium myrtillus* L. [10], and *Arctostaphylos uva-ursi* L. [14] leaf extracts. That is why we have chosen arginine as a modifying agent, hypothesizing that the combination of arginine with a plant-derived extract is a promising direction of modification of its preventive and/or therapeutic properties [3]. L-arginine supplementation with PE (PE + Arg) supported the positive effect of PE and resulted in improvement in liver lipid metabolism after IR state impact (Table 3). The previous study showed that the addition of L-arginine to plant polyphenol extracts had a positive effect on the prevention and management of IR [12].

The present results (shown in Table 3) are consistent with the results reported in the literature demonstrating the accumulation of TG, DG, and FFA in the liver of the rats with IR [57]. A decrease in PL levels in rats can occur due to an increase in phospholipase D (PLD) activity, which in turn stimulates TG accumulation [58]. In addition, CTP:phosphocholine cytidyltransferase inhibition can be stimulated in hepatocytes, leading to a decrease in PL content and TG accumulation [59]. Moreover, the maintenance of PL content could improve membrane stability and reduce lipid peroxidation reactions in hepatocytes.

Our results suggest that the administration of PE + Arg to rats resulted in a bodyweight decrease and the accumulation of TG in the liver. Moreover, blood serum Ch-HDL levels in rats showed a negative correlation with HOMA-IR. The results of our study also confirm the suppressive (dampening) effect of PE + Arg administration on the development of metabolic disorders (caused by IR) in rats. This suggests that the combination of polyphenols with L-arginine could be useful in the treatment of T2DM.

The aqueous PEO-PE gels formulated for SSE 3D printing showed a fairly (but not fully) homogeneous structure. Consequently, the 3D printing of 15% PEO-PE gels was challenging since a printing head was periodically blocked and the final lattices printed had a non-uniform structure (after visual inspection). The present 3D printing limitation was successfully resolved by decreasing the concentration of PEO and, consequently, by decreasing the viscosity of gel. Therefore, in the subsequent SSE 3D printing experiments, 12% PEO gels loaded with eumulgin as a viscosity-decreasing agent were used. Eumulgin was also found to improve the release of plant extracts in previous studies [8,33]. In summary, the aqueous 12% PEO gel was found to be a feasible base (platform) for the gels consisting of PE and PE + Arg at concentrations of 1.0 g per 10 mL. The corresponding SSE 3D-printed lattices were uniform in size and shape and of good quality (Figure 6).

The disintegration of 3D-printed preparations was investigated in vitro by placing the samples in purified water (22 ± 2 °C) and verifying by visual inspection that they were completely disintegrated within 15 min. The present SSE 3D-printed PE lattices disintegrated in vitro within 15 min, thus showing an immediate-release behavior. The aqueous PEO-PE gels (Figure 6) were also investigated for the SSE 3D printing of special round-shaped single-unit disc preparations intended for oral administration. The present 3D-printed disc preparations, with a minor modification, could be used as an immediate-release dosage form for the oral administration of cranberry leaf extract.

The present 3D-printed disc preparations, with a minor modification, could be used as an immediate-release dosage form for the oral administration of cranberry leaf extract.

## 5. Conclusions

The present study revealed the potential of phenolics found in the leaves of *V. macrocarpon* Aiton in the prevention of health issues associated with IR. The performance and efficacy of such plant-origin phenolics can be additionally augmented by their conjugation with L-arginine. Therefore, it is evident that *V. macrocarpon* leaves, which are rich in such phenolics and are the by-product of berry production, are a good candidate for the development of dietary supplements for supporting the treatment of metabolic disorders related to T2DM. The formulation of novel 3D-printed oral dosages for PE and PE + Arg is a promising way to overcome the limitations related to the oral administration of such preparations (as dietary supplements). The present study shows that one promising aqueous PEO-PE gel for SSE 3D printing is the formulation consisting of PE 100 mg/mL and eumulgin 150 mg/mL in a 12% PEO gel platform.

## Figures and Tables

**Table 1:** Phenolic compounds in cranberry leaf extracts identified by HPLC-DAD-MS.

Substances	Retention Time [min]	PE	PE + Arg
Quinic acid	1.5	+	+
ND	10.4		+
3-*O*-Caffeoylquinic acid (chlorogenic acid)	16.8	+	+
ND	26.0	+	+
(+)-Catechin	29.0	+	
*p*-Coumaroylquinic acid	29.9		+
Proanthocyanidin A type	32.9	+	
ND	33.0		+
ND	36.0	+	+
ND	37.6	+	+
Quercetin 3-*O*-galactoside	40.7	+	+
Quercetin-3-*O*-glucoside	41.8	+	+
Procyanidin dimer A2 type	42.7	+	
Quercetin-3-*O*-xyloside	43.1	+	+
Quercetin-3-*O*-arabino pyranoside	44.2	+	+
Quercetin-3-*O*-arabino furanoside	45.7	+	+
Quercetin 3-*O*-rhamnoside	46.4	+	+
Quercetin-*O*-*p*-coumaroyl hexoside-1	50.6	+	
Kaempferol 3-O-rhamnoside	51.9	+	
Quercetin-*O*-*p*-coumaroyl hexoside-2	58.2	+	+
Quercetin derivative	69.6	+	

Note: ND—a substance is not identified; “+”—a substance was found.

**Table 2:** Quantitative content of phenolic compounds and amino acids in the cranberry leaves dry extracts (mean ± SD, n = 5).

BAS Group	Spectrophotometric Method	Assay, %	
		PE	PE + Arg
Hydroxycinnamic acids	In terms of chlorogenic acid (λ = 327 nm)	11.54 ± 0.11	7.10 ± 0.07
	In terms of chlorogenic acid (λ = 525 nm); chromogenic reagent: sodium nitrite and sodium molybdate	13.59 ± 0.63	8.10 ± 0.37
Flavonoids	In terms of rutin (λ = 417 nm)	4.01 ± 0.26	2.53 ± 0.14
	In terms of hyperoside (λ = 425 nm)	4.94 ± 0.46	3.19 ± 0.45
Total polyphenols	In terms of gallic acid (λ = 270 nm)	17.16 ± 0.29	4.94 ± 0.30
	Chromogenic reagent: Folin & Ciocalteu′s Phenol Reagent (λ = 765 nm)	19.18 ± 0.43	7.59 ± 0.56
Amino acids	In terms of leucine (λ = 573 nm); chromogenic reagent: ninhydrin solution	0.88 ± 0.09	5.60 ± 0.38

**Table 3:** The effect of *V. macrocarpon* Aiton leaf extract and the corresponding extract loaded with arginine on some markers of lipid metabolism (mean ± SD, n = 6).

Indices	Experimental Groups						
	IC	IR	IR + PE	IR + PE + Arg	IR + Arg	IR + Arph	IR + Met
Blood serum							
TG, mmol/L	1.45 ± 0.19	2.35 ± 0.24 ^a^	1.69 ± 0.15 ^b^	1.58 ± 0.18 ^b^	2.15 ± 0.21 ^c^	1.75 ± 0.47	1.63 ± 0.35 ^b^
TCh, mmol/L	3.21 ± 0.19	6.99 ± 0.24 ^a^	4.09 ± 0.37 ^b^	3.57 ± 0.54 ^b^	5.44 ± 0.67 ^c^	4.95 ± 1.63 ^b^	3.38 ± 0.94 ^b^
Ch-LDL, µmol/mg protein	2.33 ± 0.45	4.67 ± 0.87 ^a^	3.09 ± 0.68 ^b^	2.12 ± 0.45 ^b^	4.02 ± 0.63	3.82 ± 0.74 ^b^	2.57 ± 0.85 ^b^
Ch-HDL, µmol/mg protein	0.99 ± 0.08	0.54 ± 0.11 ^a^	0.96 ± 0.10 ^b^	1.12 ± 0.13 ^b^	1.06 ± 0.19 ^b^	1.03 ± 0.12 ^b^	0.99 ± 0.08 ^b^
Liver homogenate							
PL, nmol/mg protein	115.7 ± 11.3	82.9 ± 7.3 ^a^	93.6 ± 8.7	108.5 ± 9.4 ^b^	90.8 ± 8.5	90.4 ± 10.7	105.9 ± 8.4 ^b^
DG, nmol/mg protein	14.23 ± 1.56	19.36 ± 1.75 ^a^	18.38 ± 2.11	16.54 ± 1.43 ^b^	17.28 ± 1.33	18.41 ± 0.96	15.81 ± 1.96 ^b^
Ch, nmol/mg protein	10.26 ± 0.96	34.28 ± 4.59 ^a^	24.31 ± 1.94	15.94 ± 2.83	29.52 ± 4.59 ^c^	27.05 ± 3.81 ^c^	19.53 ± 1.79 ^b^
TG, nmol/mg protein	57.34 ± 4.42	68.52 ± 5.17 ^a^	62.71 ± 4.25	58.47 ± 3.29 ^b^	67.41 ± 4.83 ^c^	5.91 ± 6.18	55.68 ± 3.93 ^b^
FFA, nmol/mg protein	22.83 ± 1.45	35.33 ± 2.04 ^a^	29.45 ± 1.47	25.33 ± 0.94 ^b^	30.05 ± 1.23 ^c^	27.42 ± 1.11 ^b^	24.97 ± 1.73 ^b^

a—indicates a significant difference relative to the IC group (*p* ≤ 0.05). b—indicates a significant difference relative to the IR group (*p* ≤ 0.05). c—indicates a significant difference relative to IR + PE + Arg group (*p* ≤ 0.05).

## Data Availability

Not applicable.
